# The Cognitive Changes Among Patients over 65 Years of Age in a Rural Area—The Preliminary Report of Protective and Predisposing Factors

**DOI:** 10.3390/neurolint17110180

**Published:** 2025-11-03

**Authors:** Radoslaw Zachara, Daria Gendosz de Carrillo, Adam Wlaszczuk, Agnieszka Gorzkowska, Wiktoria Mazur, Halina Jedrzejowska-Szypulka

**Affiliations:** 1 Department of Physiology, Faculty of Medical Sciences in Katowice, Medical University of Silesia in Katowice, 40-752 Katowice, Poland; dgendosz@365.sum.edu.pl (D.G.d.C.); awlaszczuk@365.sum.edu.pl (A.W.); hszypulka@365.sum.edu.pl (H.J.-S.); 2 Department of Histology and Cell Pathology, Faculty of Medical Sciences in Zabrze, Medical University of Silesia in Katowice, 41-808 Zabrze, Poland; 3 Department of Neurology, Faculty of Health Sciences in Katowice, Medical University of Silesia in Katowice, 40-752 Katowice, Poland; agorzkowska@sum.edu.pl; 4 Student Science Club of Engineering and Systems Biology, Biotechnology Centre, Silesian University of Technology, 44-100 Gliwice, Poland; wm312874@student.polsl.pl; 5 Faculty of Biomedical Engineering, Silesian University of Technology, 41-800 Zabrze, Poland

**Keywords:** arterial hypertension, diabetes mellitus, dementia, mild cognitive impairment, aging, amyloid, APOE

## Abstract

**Background:** Aβ1-42 and APOE concentrations, as well as Aβ42/40 ratio, may be considered as a link between hypertension (HTN) or diabetes mellitus (DM), brain amyloidosis, and dementia. HTN and DM are associated with cognitive impairment and may contribute to the development of Alzheimer’s disease (AD). This preliminary study aimed to evaluate the impact of vascular risk factors on the concentration of biochemical AD markers and cognitive state. As it is a cross-sectional study in nature, causal relationships cannot be established. **Methods:** The study was conducted in the south of Poland among a rural population over 65 years of age. A total of 58 patients qualified into the study were divided into groups according to the presence of HTN (*n* = 18) or HTN coexisting with DM (*n* = 40). A healthy control group was also formed (*n* = 20), resulting in 78 study participants. The study population was also divided based on M-ACE results, forming a normal cognition group (NC) and a deteriorated cognition group (DC). Biochemical tests, neurological scales assessments, and ultrasound examinations were conducted. **Results:** Patients who scored in the normal range on the M-ACE had higher Aβ1-42 (median 38.52 vs. 27.35 pg/mL, *p* = 0.02) and apoE concentrations (median 125.0 vs. 65.73 μg/mL, *p* = 0.002), and a higher Aβ42/40 ratio (median 0.39 vs. 0.29 *p* < 0.000) compared to the DC group. Considering the study groups, the highest Aβ42/40 ratio was found among the HC group (median 0.47). The median score for the M-ACE scale was 3 points lower when HTN and DM coexisted, compared to the sole diagnosis of HTN (25 points and 28 points, respectively). A higher number of years of education correlated with better M-ACE results. Lipid and uric acid concentrations were not related to M-ACE or MMSE scores. An inverse relationship connected Aβ1-40 and Aβ1-42 to BMI, the duration of HTN treatment, and glycated hemoglobin. **Conclusions:** Aβ1-42, APOE, and Aβ42/40 are not only correlated with cognition but also related to patient’s disease profile. The coexistence of DM and HTN was associated with the most significant decline in cognitive functioning. However, a higher number of years of education may protect against the development of dementia in old age. The roles of cholesterol and uric acid in cognitive decline are still inconclusive.

## 1. Introduction

The prevention and treatment of dementia is one of the greatest challenges in the modern world, and Alzheimer’s disease (AD) is the most common cause of it [1]. AD is estimated to affect nearly 7 million people in the European Union [2]. The most well-known hypothesis of AD development is related to beta-amyloid (Aβ) metabolism. The amyloid precursor protein (APP) cleavage can be conducted by α-secretase and γ-secretase (the non-amyloidogenic pathway), which does not produce Aβ, or by β-secretase and γ-secretase producing cleavage fragments and finally, Aβ1-42 and Aβ1-40 [3,4,5]. In physiological conditions, Aβ is decomposed by neprilysin, insulin-degrading enzyme (IDE), cathepsin B, or alternatively transported through the blood–brain barrier [6,7]. The development of AD is characterized by the elevated production of soluble Aβ, its decreased degradation, and, finally, its formation of insoluble senile plaques initially deposited in neocortex [6,7]. These plaques initiate inflammation, resulting in synaptic dysfunction [8]. AD can be genetically determined (EOAD, early onset AD) or induced by other causes, such as environmental factors and the aging process (LOAD, late onset, sporadic AD) [6]. Aβ physiological roles influence antioxidation processes—especially copper (Cu^2+^) and iron (Fe^3+^) ions reduction, blood–brain barrier permeability regulation, and memory consolidation [4]. Aβ can activate α7-nicotinic acetylcholine receptors (α7-nAChRs) and regulate long-term potentiation (LTP), influencing memory functions and learning [4]. α7-nAChRs may have an important role in Aβ1-42 internalization and intracellular accumulation [9]. Thus, nAChRs are correlated with AD progression [10].

The apolipoprotein E (APOE) ε4 allele is considered to be the most significant genetic risk factor for late-onset AD [11]. It is associated with increased Aβ plaque accumulation [12,13]. The APOE4 allele is characterized by the highest Aβ deposition compared to APOE2 or APOE3, resulting in the highest relative risk of AD development [4]. One copy of the APOE4 allele elevates the relative risk (RR) of developing AD 3.7-fold compared to the APOE3 homozygote, and a relative risk for the APOE4 homozygote is 12-fold higher [11]. The APOE genotype is associated with serum apoE concentration [14]. The mechanism of APOE4 influence on Aβ metabolism may be the result of impaired Aβ clearance, increased activation of microglia propelling inflammation processes resulting in neurotoxicity, and further Aβ accumulation [4]. ApoE may also regulate the blood–brain barrier (BBB) function [11,13,15].

Neuroinflammation and the function of microglia and astrocytes are important factors in AD pathogenesis. The role of inflammation in AD is complicated and possibly diversified in different AD stages [16]. Initially, inflammation stimulates Aβ clearance, but persistent exposure to Aβ induces tolerance and impairs the microglial cells’ functions [17]. The astrocytes also participate in Aβ metabolism, and their function depends on APOE, connecting immunological and metabolic factors [18]. Aβ1-42 stimulates the production of cytokines (i.e., Il-1β, Il-6), propelling neuroinflammation and suppressing synaptic transmission even before structural changes [17]. Aβ1-42 is considered to be more pathogenic than Aβ1-40 due to its higher fibrillation potential [19,20]. There are articles indicating the correlation and reflection between CSF and the concentrations of Aβ1-40 and Aβ1-42 in blood [21,22].

Considering dementia prevention, we focused on the patient’s disease profile and its influence on cognitive decline. Vascular dementia occurs in 15% to 30% of dementia cases and is the second most common dementia type after AD [23]. The population of individuals aged 65 and older is often treated for more than one chronic disease. A total of 36% of Europeans aged 65 and above receive treatment for at least two chronic conditions. In Poland, this percentage is even higher, at 56% [24]. Approximately 1.39 billion adults worldwide had hypertension (HTN) in 2010. More recent data considering the global prevalence of HTN were not identified. The research unequivocally proves there is a link between HTN and dementia, as well as with mild cognitive impairment (MCI), particularly in patients with uncontrolled HTN [25]. Numerous scientific reports describe the impact of HTN on cognitive functions [26,27,28]. It is believed that up to 30% of patients diagnosed with HTN may suffer from MCI [29], and controlling HTN may improve cognitive functioning [30,31]. There is also evidence suggesting that antihypertensive treatment may prevent the occurrence of cognitive impairment [32]. HTN can impair cognitive functioning in many mechanisms and lead to neuron loss due to vascular damage, changes in white matter (leukoaraiosis), damage to neurovascular units, reduced blood flow through the brain, damage to the blood–brain barrier, perivascular damage, or proteinopathy [28]. Systemic and brain renin–angiotensin system (RAS) dysfunctions are connected to HTN development [33]. In the brain, RAS modulates the release of norepinephrine, glutamate, GABA, acetylcholine, vasopressin, and possibly dopamine, influencing the brain functions [34]. Moreover, angiotensin II affects mitochondrial functioning, reducing energy production and increasing reactive oxygen species (ROS) generation. Next, ROS damage neurons [35]. Elevated blood pressure causes high shear stress, intensifying the inflammatory process and arteriosclerosis, promoting further endothelial damage [36]. This leads to the remodeling of the vessel wall and a reduction in the range of vascular flow regulation [37]. Changes in blood flow in small brain vessels contribute to lacunar infarcts, leukoaraiosis, and brain hemorrhages [38]. Chronic HTN disrupts the functioning of the blood–brain barrier [39] and substances that pass through it—mainly proteins in the perivascular space, which can initiate and intensify the inflammatory process [40].

DM pathology affects the cardiovascular system, facilitating the development of HTN. DM is complicated by HTN development in up to 74% of patients, and it is more frequent in older populations [41]. DM may affect cognitive functions. We assessed the influence of the coexistence of HTN and DM in our study, because the sole occurrence of DM in the geriatric population is rare. In 2021, there were 529 million people worldwide suffering from diabetes mellitus (DM), and it was estimated that by 2050, there will be 1.31 billion diabetics [42]. There are many mechanisms in which DM contributes to central nervous system damage, including the involvement of DM-enhanced small vessel disease, the presence of advanced glycation products which damage neuronal mitochondria, and recurrent episodes of hypoglycemia [43,44,45]. An association between DM and MCI, as well as dementia, was observed [46]. There is existing data showing a 60% higher risk of developing dementia in diabetics compared to individuals not treated for DM, and a higher risk among women than men [47]. Treating DM can reduce the risk of developing dementia [48]. The younger age of DM onset is statistically associated with a higher risk of dementia development [49]. The level of glycated hemoglobin, as a marker of glycemic control, is higher in patients with mild dementia [50]. However, the elevated blood glucose levels can be a risk factor for dementia even in nondiabetics [51]. According to studies performed on the American population, HTN occurs in up to 78% of adult diabetics, leading to overlapping mechanisms affecting central nervous system damage in both conditions [52]. The cumulative effect on the vascular burden of neurovascular–glial unit resulting in perfusion abnormalities is especially emphasized [53]. Medical literature also discusses the impact of cholesterol on cognitive functions [54,55,56]. The role of uric acid concentration and its influence on AD and vascular dementia remains a topic of ongoing debate [57,58].

The novelty of our work stems from the combination of parameters studied and the rural population examined. Based on current knowledge and the literature study, we found an important gap, which led us to the aim of our study, which is to assess the simultaneous influence of vascular risk factors and Aβ1-40 and Aβ1-42, and apoE concentrations on cognition in a specific rural population. The protective effect of education was also considered. In our work, we try to identify cost-effective (after large-scale implementation of this method) and widely available approaches for the early assessment of blood biomarkers related to cognitive impairment. We also evaluated the impact of optimal management of hypertension and diabetes on the risk of developing cognitive decline (Spearman correlations: amyloid and glycated hemoglobin, amyloid, and intima–media complex).

We would like to underscore that assessing cognitive functioning within a rural primary care population provides unique insights. The majority of our study participants are physically active farmers, with 78.2% reporting over 150 min of physical activity per week—higher compared to the rate of 50% of the population over 60 years of age performing physical exercise for more than 150 min per week [59]. Most of the research published in this area focuses on urban populations, while our study delivers valuable input from a rural setting that is underrepresented in the literature.

## 2. Materials and Methods

**Figure 1:**
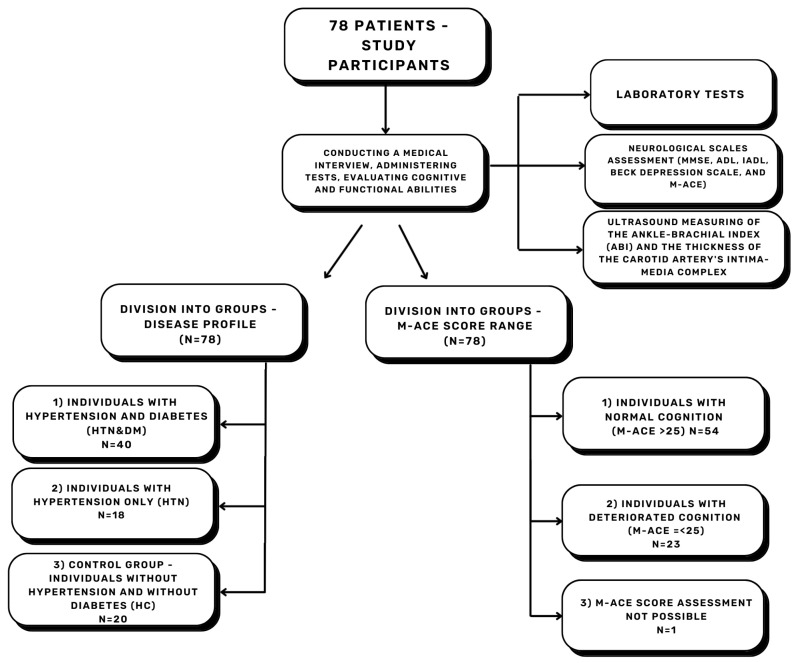
The study overview flowchart. N—quantity, M-ACE—Mini–Addenbrooke’s Cognitive Examination, MMSE—Mini–Mental State Examination, IADL—Instrumental Activities of Daily Living, ADL—Basic Activities of Daily Living, HTN—hypertension, DM—diabetes mellitus, HC—healthy controls.

This preliminary study was conducted on the population of a rural health center: Non-Public Health Care Institution REMED (NZOZ REMED). Compared to other studies, the rural study population distinguishes our research. NZOZ REMED is located in Poland, in the municipality of Borzęcin, in the Małopolskie Voivodeship. The population of the municipality was 8088 residents [60]. Ethical approval for the study was obtained from the Bioethics Committee—resolution no. PCN/CBN/0022/KB1/123/21 granted on 16 November 2021. A total of 80 patients were considered for participation in the study; finally, 78 patients were included, and 2 patients were excluded (details below). Individuals were selected based on ICD-10 (International Classification of Diseases, version 10) diagnoses from their long-term medical records. The inclusion criteria for the study were 65 years of age or older, diagnosed with HTN or DM, and the absence of any exclusion criteria. The healthy control (HC) group was composed of patients over 65 years of age without diagnosis of DM or HTN and not meeting exclusion criteria. Moreover, HC group patients were tested for HTN (measurement of blood pressure using Riva–Rocci–Korotkow method) and DM (fasting glucose and glycated hemoglobin) to exclude these diseases. The exclusion criteria included psychiatric disorders in the medical history—diagnoses from the “F” group according to ICD-10; we allowed tobacco and caffeine addictions, sleep disorders, cognitive function disorders, dementia, and the presence of nervous tics. One person was excluded due to depression, and the second one due to the diagnosis of ischemic stroke. The categorization of patients reflected the most common disease profile in primary care (Figure 1).

Upon analyzing the data, we identified notable differences between patients with normal cognition (NC; M-ACE > 25) and patients with deteriorated cognition (DC; M-ACE ≤ 25) within the entire study group. To accurately assess the data, we divided the patients into NC and DC groups based on their M-ACE results (Figure 1). One patient was excluded due to hearing and vision impairment, which precluded M-ACE or MMSE assessment.

The study overview flowchart is presented in Figure 1.

The study group data is included in Table 1.

Medical interviews were conducted with each patient and included questions related to addiction history, education, social relationships, and medical history. A total of 15 milliliters of blood were collected from study participants for biochemical tests. In blood plasma, the following tests were performed: complete blood count and measurement of glycated hemoglobin concentration. In blood serum, the following parameters were assessed: lipid profile (enzymatic method), uric acid concentration (enzymatic method with uricase and peroxidase), alanine aminotransferase (ALAT) activity (kinetic method), and creatinine concentration (kinetic method with picric acid in an alkaline environment) in a certified diagnostic laboratory. Complete blood count was measured with the SYSMEX XN 2000 hematological analyzer (Sysmex Corporation, Kobe, Japan), and all other blood parameters were tested using the ALINITY CI immunochemistry analyzer by ABBOTT (Chicago, IL, USA). Other biochemical parameters were measured using dedicated tests:

(1) Amyloid 1-42 High Sensitive ELISA Kit for Amyloid Beta Peptide 1-42 (Ab1-42) HEA946Hu
(2) Amyloid 1-40 ELISA Kit for Amyloid Beta Peptide 1-40 (Ab1-40) CEA864Hu
(3) APOE SEA704Hu

Cognition and functioning were assessed using the following scales:

- MMSE (Mini–Mental State Examination)—a screening tool for cognitive assessment. Scores range from 0 (severe cognitive decline) to 30 (normal cognition) [61].
- ADL (Basic Activities of Daily Living)—an instrument assessing functional capacity in basic activities, for example, eating, dressing, and continence. Scores range from 0 (dependent patient) to 6 (independent patient) [36].
- IADL (Instrumental Activities of Daily Living)—evaluation of functional capacity in more complex activities, such as independent shopping and economic management. Scores range from 8 (dependent daily living functioning) to 24 (independent daily living functioning) [62].
- Beck Depression Inventory—used for depression diagnosis. The version with 21 questions was used. Scores range from 0 (no depression symptoms) to 63 (severe depression symptoms) [63].
- M-ACE (The Mini–Addenbrooke’s Cognitive Examination)—a screening tool for dementia diagnosis. The minimum score is 0 (indicating dementia), and the maximum score is 30 (normal cognitive level). The recommended cut-off used was 25 and 21 points [64]. We used 25 points to divide the study group into the NC group (normal cognition) and the DC group (deteriorated cognition).

Ultrasonographic examination of patients included in the study was performed to measure the ankle–brachial index (ABI) [65]—the range 0.9–1.15 was considered normal. Also, the thickness of the carotid artery intima–media complex [66,67] (using the Alpinion E-CUBE 7 device, Alpinion Medical Systems Co., Ltd., Seoul, Republic of Korea) was assessed. The intima–media complex thickness at the level of the common carotid artery is considered to be a marker for blood pressure stabilization [68]—the normal thickness was considered to be 1 mm or less.

Statistical analysis was conducted using Statistica 13. The normality of data distribution was verified using the Shapiro–Wilk test. In cases where normality was confirmed, homogeneity of variance was assessed using Levene’s test, and depending on the number of groups compared, followed by Student’s t distribution test or analysis of variance (ANOVA) for independent samples. Post hoc analysis was performed based on the NIR test. When normality was not confirmed, the Wilcoxon test, the U Mann–Whitney test, or the Kruskal–Wallis rank analysis of variance was conducted for independent samples, depending on the number of groups compared and group characteristics. Associations between quantitative variables were evaluated using Spearman’s rank correlation coefficient for subgroups defined by M-ACE values.

## 3. Results

The population of our study was living in a village. The majority of the population were farmers. This distinguishes our study as unique. In our study, there were 28 male and 50 female patients. The results of highest statistical significance between male and female study participants were the duration of smoking—median value for men was 23.75 [years] and 0 [years] for women (*p* < 0.000); median HDL −1.01 [mmol/L] for men and 1.28 [mmol/L] for women (*p* < 0.000); median TG 1.63 [mmol/L] for men and 1.23 [mmol/L] for women (*p* = 0.043); mean uric acid 383.65 [μmol/L] for men and 280.9 [μmol/L] for women (*p* = 0.001). It is important to mention that 78.2% of participants were physically active for at least 30 min daily. This percentage is higher compared to the rate of 50% of the population over 60 years of age performing physical exercise for more than 150 min per week [59]. Many of the participants used bicycles for transportation or walked. In addition, residents own gardens or small farms, which resulted in long times spent on activity. A total of 11.5% of participants were physically active for at least 30 min three times a week. A total of 7.7% of participants were less active but not inactive. Only one patient was mainly inactive.

Detailed results of neurological scales, ultrasonographic, and biochemical tests in different treatment groups are included in the Supplementary Materials (Table S2).

**Figure 2:**
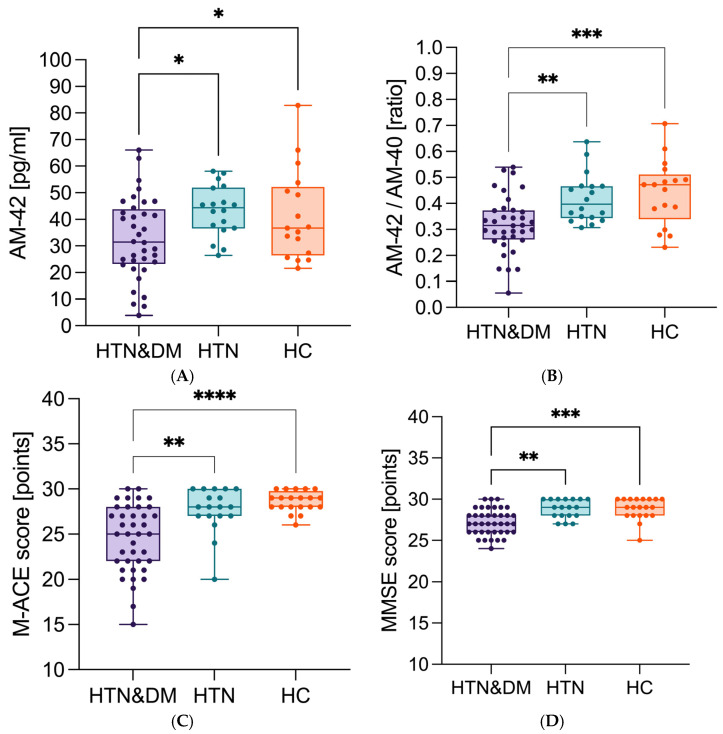
The association between (**A**) Aβ1-42 concentration, (**B**) Aβ42/40 ratio, (**C**) M-ACE, (**D**) MMSE, and the study groups (the min–max values presented with all points); HTN—hypertension, HC—healthy controls, M-ACE—Mini–Addenbrooke’s Cognitive Examination, MMSE—Mini–Mental State Examination; detailed values with confidence intervals are provided in the Table S1. * *p* < 0.05, ** *p* < 0.01, *** *p* < 0.001, **** *p* < 0.0001.

Considering the disease profile, in the HTN–DM group, the concentration of Aβ1-42 (median 32.8 pg/mL) was the lowest (Figure 2A). The differences were statistically significant, considering HTN *p* = 0.015 and in relation to HC *p* = 0.047. Similarly, the Aβ42/40 ratio (median 0.32) (Figure 2B) was lowest in the HTN–DM group and the differences were statistically significant in comparison to the HTN group *p* = 0.002, and in relation to HC group *p* < 0.000. Moreover, the HTN–DM group was characterized by the lowest M-ACE (median 25 points) (Figure 2C) (vs. HC *p* < 0.000 and vs. HTN *p* = 0.006) and MMSE (median 27 points) (Figure 2D) (vs. HC *p* < 0.000 and vs. HTN *p* = 0.002).

**Figure 3:**
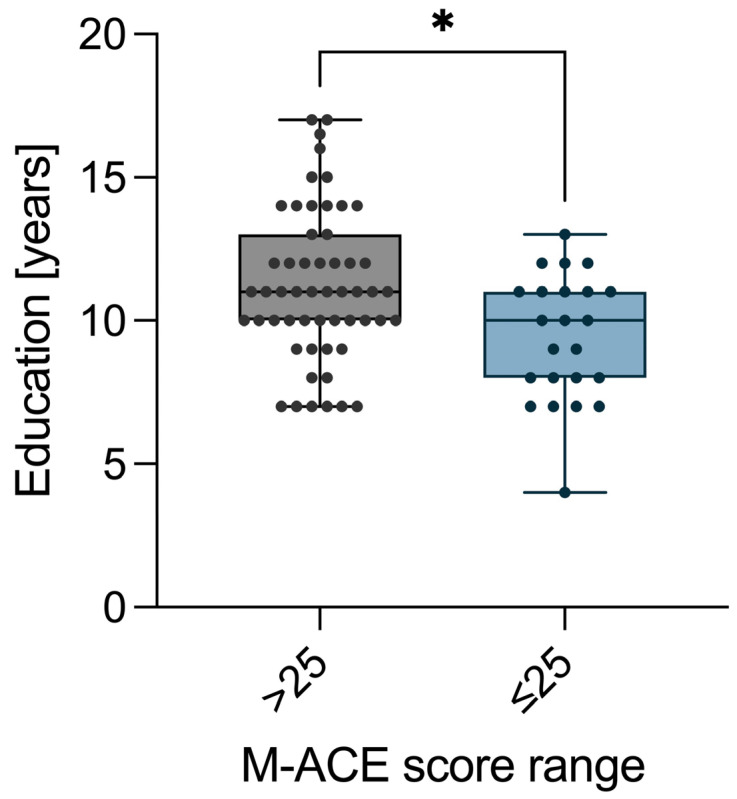
The association between years of education and M-ACE score range. * *p* < 0.05.

Education level (years of education) is associated with cognitive functioning (Figure 3), because we found that the median number of years of education was higher among the NC patients (median value 11) compared to the DC patients (median value 10) (*p* = 0.015) (Table 2).

Patients in the DC group were characterized by shorter education time (median 10 vs. 11 years, *p* = 0.015), longer time of lipid-lowering treatment (considering patients on lipid-lowering therapy, median 14 vs. 10 years, *p* = 0.04), thicker IMC (median IMC L 1.1 vs. 0.9 mm, *p* = 0.005; IMC R median 1.05 vs. 0.9 mm, *p* = 0.01), lower Aβ1-42 concentration (median 27.35 vs. 38.52 pg/mL, *p* = 0.02), lower Aβ42/40 ratio (median 0.29 vs. 0.39, *p* < 0.000), and lower apoE concentration (median 65.73 vs. 125.0 μg/mL, *p* = 0.002) (Table 2).

**Figure 4:**
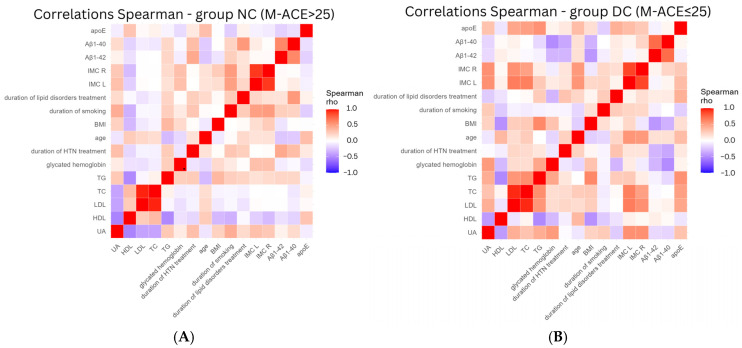
Spearman’s rank correlation coefficient in study parameters, (**A**) NC—normal cognition group; (**B**) DC—deteriorated cognition group; apoE—apolipoprotein E; Aβ—beta-amyloid; IMC R—intima–media complex thickness right side; IMC L—intima–media complex thickness left side; BMI—Body Mass Index; TG—triglycerides; TC—total cholesterol; low-density lipoproteins; high-density lipoproteins, UA—uric acid.

Analyzing correlations between studied parameters among DC patients (Figure 4B), the notable inverse relationship with Aβ1-40 and Aβ1-42 was found for BMI (−0.335 and −0.448, respectively), duration of HTN treatment (−0.377 and −0.316, respectively), and glycated hemoglobin (−0.495 and −0.316, respectively). Thus, patients with higher BMI, longer duration of HTN treatment, and higher glycated hemoglobin concentration were characterized by decline in Aβ1-40 and Aβ1-42 concentrations. IMC L and IMC R (HTN stabilization markers) were more strongly positively related to age (0.543 and 0.522, respectively), TC (0.644 and 0.488, respectively), LDL (0.656 and 0.566, respectively), and APOE (0.345 and 0.283, respectively) among DC patients. In some values, we noted the change in direction depending on cognition status. The concentrations of Aβ1-40 and Aβ1-42 decreased during aging among NC patients (Figure 4A) (−0.225 and −0.245, respectively) and increased among DC patients (0.161 and 0.164, respectively).

## 4. Discussion

Low Aβ1-42 concentration is considered to be associated with AD development [69,70]. It is believed that brain amyloid deposition and sequestration in plaques [71] may result in its lower blood concentration [72]. Not only the diagnosis but also the progression of AD from mild to severe resulted in decreased levels of blood beta-amyloid [73]. Concerning Aβ1-42, our results are consistent with other observations. We found lower levels of Aβ1-42 in the DC patients, possibly indicating developing cognitive decline. Comparing the HTN–DM group to the HTN and HC groups, we found that the levels of Aβ1-42 were the lowest among HTN–DM patients, suggesting the probable impact of multimorbidity on the process of AD development.

The reduction in Aβ42/40 ratio is considered an important marker for MCI and prodromal stages of AD [74], and is related to a 70% increase in the risk of progression from MCI to AD within 2 years [75], with visible FDG-PET Aβ brain depositions [76,77]. The increase in the Aβ42/40 ratio (approximately 1:9) is associated with suppression of the Aβ1-42 nucleation, exerting a neuroprotective effect in brain [20]. We found in our study that in the HTN–DM group, the Aβ42/40 ratio was the lowest compared to the HTN and HC groups, implying these patients were at the highest risk of developing brain amyloidosis and dementia [77].

Following the assessment of Aβ influence on cognition, we attempted to observe the influence of other factors on blood Aβ concentration. Depending on the research, age has been reported to be negatively [78] or positively [79] correlated with Aβ1-42 in the study population, regardless of age. However, no specified correlation was found for patients aged 65 and over [79]. In our study, the influence of age on Aβ1-42 and Aβ1-40 depended on the cognitive status; among NC patients, the tendency was negative; among DC patients, it was slightly positive, possibly mirroring the effect of developing dementia.

Authors point to positive [80] as well as negative correlations [81] in considering BMI and Aβ1-42 or Aβ1-40 concentrations. A high-fat diet in mice, which may reflect the influence of obesity, resulted in increased plasma Aβ1-42, endothelial dysfunction, vasoconstriction, and increased blood pressure [82]. APP, Aβ1-42, and Aβ1-40 were also found in body fat tissue, pointing to adipose tissue as an important source of Aβ1-42 in obese patients [83]. In our study, BMI was inversely related to Aβ1-42 and Aβ1-40 among DC patients, suggesting the influence of obesity on the development of cognitive impairment. Adipose tissue may be the source of Aβ1-42 and Aβ1-40, but higher BMI may also be partially affected by higher lean mass (muscle, bones), especially among farmers. As long as the course of serum concentration of Aβ1-42 and Aβ1-40 is not fully understood, further research is needed.

The low apoE concentrations were correlated with dementia, cognitive impairment, and hippocampal size reduction [14,84]. Lower apoE levels were related to APOE4 allele presence and the lowest concentrations indirectly suggested ε4/ε4 genotype [14]. Similarly, the highest apoE concentrations were observed among patients diagnosed with normal cognition in M-ACE (Table 2). The data presented in other articles confirms our observations [85]. In our study, apoE was not related to IMC L and IMC R among NC patients, while it was positively related among DC patients. This dependence may be the result of the influence of LDL and TC on IMC. IMC was measured on the level of common carotid artery, where it should reflect the pathological changes in the course of HTN, but the synergistically harmful effects of hypercholesterolemia are also possible [68]. Additionally, the duration of lipid disorders treatment was positively associated with apoE among DC patients.

No statistically significant differences were found for Aβ1-42, the Aβ42/40 ratio, MMSE, and M-ACE scores between HTN and HC. Considering Aβ42/40 (median value HC 0.47 and HTN 0.40) and M-ACE (median value HC 29 and HTN 28 points) scores, the expected trend is noticeable. It is consistent with findings from larger studies, suggesting that the differences would be more evident in larger cohorts. We expected Aβ1-42 concentration to be the highest among the HC group (median value 41.4 pg/mL), but the concentration is slightly higher among the HTN group (median value 43.3 pg/mL), possibly due to the limited quantity of patients. As it is a preliminary report, the sample size is our limitation. The differences may be more pronounced in our future observations.

Concerning disease profile, we aimed to observe and explain the mechanisms of neuronal damage in HTN and DM. Concerning HTN fluctuations in blood pressure, this causes the remodeling of the vascular wall and the deterioration of brain perfusion [28]. This results in a reduction in blood flow within the temporal and occipital lobes and a decrease in the thickness of the cerebral cortex [86]. The impact of blood pressure on cognitive functioning depends on age. High blood pressure in middle age, but also low blood pressure in old age, can negatively affect the cognitive functioning of the patient, as high pressure in old age can compensate for the reduction in the lumen of vessels by atherosclerotic changes [28,87]. The cerebral blood flow does not depend strictly on blood pressure. Studies revealed that more restrictive control of systolic pressure, i.e., maintaining its value below 120 mmHg, increases cerebral perfusion compared to maintaining systolic pressure below 140 mmHg [88]. Stabilizing the blood pressure at the level of 130 mmHg or less from the age of 40 years is considered to reduce the possibility of developing dementia [89]. There are numerous references in the literature linking the pathogenesis of AD with HTN [90,91,92]. Small vessel disease, microinfarcts, and microhemorrhages intensify atherosclerotic processes within the cerebral circulation [93]. According to studies, the expression of angiotensin II deteriorates cognitive functioning and reduces synaptic plasticity through the expression of p38MAPK (mitogen-activated protein kinase p38) [94]. The gene expression involved in the production of beta-amyloid in the hippocampus area also changes [95]. The loss of the blood–brain barrier integrity, oxidative stress, and the presence of free radicals trigger the microglia activation, which intensifies the inflammatory process, ultimately leading to the death of neurons [96].

Considering HTN stabilization markers in our study, the median IMC L was 0.2 mm higher, and IMC R 0.15 mm higher in DC patients in comparison to NC patients. In the HTN–DM and HTN groups, above-normal IMC was measured in patients with lower MMSE results (Figure S3) It could point to the influence of blood pressure normalization on cognitive functioning. The inverse relationship between the MMSE result and IMC R confirms this relation. The results for attention and ability to count, as a MMSE scale component, mostly affected the total results of MMSE (Table S2). Our observations are consistent with data in the global literature [28,29,31]. It possibly indicates the primary dysfunctions caused by unstable HTN.

Concerning Aβ1-42 and Aβ1-40, the HTN may activate receptors for glycation end (RAGE) products, exacerbating the deposition of amyloid plaques [97]. Our observations concerning HTN treatment time are differentiated, and it may reflect in the course of developing dementia.

Considering the additional HTN influence of DM on cognitive decline, hypoglycemia, which is most often associated with the use of insulin or sulfonylureas [98], mainly affects patients with the worst glycemic stabilization and the most profound cognitive decline. It intensifies reactive oxygen species production, blood–brain barrier leakage, and consequently, neuronal death [44]. A single episode of severe (requiring hospital care) hypoglycemia can double the risk of dementia [99]. DM-enhanced small vessel disease causes clinically silent brain microstrokes, impairing cognitive functions [100]. High glucose concentration intensifies the formation of advanced glycation products (AGEs), which reduce nitric oxide synthesis and damage mitochondria, causing the formation of free radicals [101]. AGEs modify the expression of the transcription factor PDX-1, which participates in the regulation of insulin secretion [102,103]. An increase in DPP-4 activity—one of the enzymes involved in incretin metabolism and regulating glucose concentration, was associated with lower scores in the Montreal Cognitive Assessment Scale (MoCA) [104]. Amylin, which is secreted with insulin from pancreatic beta cells [45], presumably impacts the pathology of AD, being a component of beta-amyloid conglomerates [45]. Both insulin and beta amyloid are degraded by the insulin-degrading enzyme (IDE); thus, hyperinsulinemia present in DM patients may competitively affect the increase in beta amyloid accumulation [44,105]. The deterioration of the glymphatic system functioning in DM patients reduces the effectiveness of removing beta amyloid from the central nervous system [106]. There is a larger number of receptors for insulin-like growth factor (IGF-1R) in the temporal lobes of the brains of people suffering from AD [107]. Gontier et al. showed that in tamoxifen-induced neuronal IGF-1R knock-out mice [108], lower intensity of inflammatory processes and reduced load of beta-amyloid plaques were presented. The mice also performed better in functional tests. In our study, no statistically significant associations were found between diabetes control in HTN–DM and M-ACE scores. The participants of our study were above 65 years of age, which, in the case of the HTN–DM group, resulted in a prolonged period of diabetes treatment and exposure to recurring episodes of hypoglycemia in the past. The episodes of hypoglycemia may lower the concentration of glycated hemoglobin. The relation between Aβ1-40, Aβ1-42, and glycated hemoglobin may indicate the pathological mechanism. In larger study groups, the correlation between the increase in glycated hemoglobin concentration and cognitive impairment is emphasized [109,110]. Even a one percent increase in the level of glycated hemoglobin correlates with a decrease in the DSST scale score by 1.75 points and 0.20 points in the MMSE [111]. The analysis revealed the inverse relationship between glycated hemoglobin and Aβ1-42 (−0.316), or with Aβ1-40 (−0.495) in DC patients, which signifies that worse DM control is associated with the increase in concentration of markers connected to cognitive decline in AD.

Considering the M-ACE and MMSE scales, the lowest results were achieved by patients treated for both HTN and DM. The median score for the M-ACE scale was 3 points lower when both diseases coexisted, compared to the diagnosis of HTN only. Verbal fluency as a component of M-ACE and recall in the MMSE scale had the greatest impact on the final test result, indicating initial cognitive deficits (Supplementary Materials). Verbal fluency tests are considered as the marker anticipating cognitive decline among amnestic MCI patients [112].

High lipid concentration may affect the processes deteriorating cognition in HTN and DM. According to the literature, the lipid concentration and cognition may be influenced by blood pressure and sex [113]. Moreover, HDL is positively correlated with higher MMSE result and the statin use is possibly connected to a reduced risk of incidence of AD [114]. In the study by Solomon et al., elevated total cholesterol levels in middle age increased the risk of developing AD and vascular dementia [55]. Solomon also suggested the influence of hypolipidemic treatment on improving episodic memory and better results in psychomotor memory tests [30]. However, high cholesterol concentration in old age is not significantly correlated with the state of cognitive functioning [115]. These reports underline the importance of controlling lipid parameters in middle age [115]. In the COSMIC study, data from 20 cohorts, comprising a total of over 48,000 patients from 15 countries, were used [116]. Higher cholesterol levels were recognized as a protective factor, reducing the risk of cognitive disorders. This relationship was demonstrated for the Caucasian race, but not for Asians, in whom having higher cholesterol concentration presented more intense cognitive function disorders. In the Aspirin in Reducing Events in the Elderly (ASPREE) study, it was indicated that very high HDL cholesterol levels (>80 mg/dL) increase the risk of dementia by 27% compared to the concentration of the HDL-C 40–60 mg/dL range [117]. In summary, there are some controversies, but the most recent data suggests the influence of high LDL cholesterol levels in midlife on the development of dementia. In our study, no statistically significant differences were found between M-ACE or MMSE results and lipid concentration. Aβ1-40 was more strongly positively associated with the duration of lipid disorders treatment among NC patients and apoE was more strongly positively associated with the duration of lipid disorders treatment among DC patients. These observations might be of the effect of statins’ use, but further research in this area is needed [118,119].

Uric acid is a product of purine metabolism from the degradation of DNA and RNA [120]. Hyperuricemia—elevated uric acid levels in the blood (above 6.8 mg/dL), is associated with an increased risk of cardiovascular diseases, including HTN [121]. The association of hyperuricemia with cognitive functioning is complex, perhaps due to both its pro- and anti-oxidant properties [122]. The concentration of uric acid in the cerebrospinal fluid corresponds to its concentration in the blood and with the functioning of the blood–brain barrier [123]. The potential role of elevated uric acid levels in AD prevention is emphasized [124]—a reduction in occurrence by even 31% [122]. Hypouricemia (uric acid concentration below 4.91 mg/dL) is associated with an increased frequency of AD [125]. Increased uric acid levels may reduce the risk of developing AD and Parkinson’s disease, at the same time increasing the risk of developing vascular dementia [57,58]. In some studies, no effect of uric acid concentration on cognitive functioning was found for the entire population studied [120]. In our study, we did not find a statistically significant correlation between uric acid concentration and MMSE or M-ACE. However, the uric acid concentration differed significantly between the HTN–DM and HC groups (Supplementary Materials). High uric acid concentration is associated with metabolic syndrome and its components, i.e., HTN, hypertriglyceridemia, and glucose concentration, which are connected to DM [126,127]. It may present the influence of comorbidity on purine metabolism. In the HTN–DM group, 52.5% of patients were obese—more than in any other group. Moreover, 5% of patients in the HTN–DM group were obese in class III. The relation between UA and the duration of lipid disorders treatment may be explained by the lowering of UA concentration by statins and possibly patients’ adherence to therapy [128]. The association between UA and glycated hemoglobin, especially among DC patients (0.516), reflects the influence of metabolic syndrome and its components on hyperuricemia.

Education level is considered one of the most important potentially modifiable risk factors for dementia development [89]. The duration of education time may contribute to an increase in cognitive reserve and preservation of sufficient total brain volume for a longer time [129]. Mukadam et al. found that dementia incidence declined in high-income countries (e.g., the USA and European countries), suggesting that, among other factors, a longer education time may help preserve cognition [130].

In the meta-analysis conducted by Mollalo et al. [131], which included data from 19 countries, a higher prevalence of dementia was observed in rural populations. It was suggested that rural residents may experience greater social isolation and possess lower levels of education, whereas urban inhabitants are more exposed to air pollution and experience higher levels of stress. The difference in dementia prevalence between rural and urban populations was smaller in countries with higher levels of education and higher income. Portugal was the only European country included in the analysis, with the majority of data derived from studies conducted in Asia. Living conditions in rural areas vary across different regions of the world. Research on this topic remains limited in European studies, and the health issues faced by rural and urban populations differ. Hence, there is further a need to investigate the situation of the rural population in Poland.

As it is a cross-sectional study in nature, causal relationships cannot be established.

## 5. Conclusions

Our preliminary study showed that Aβ1-42, APOE, and Aβ42/40 blood concentrations are correlated with cognition state. Variations in Aβ1-42 and Aβ42/40 concentrations among the study groups point to a correlation between HTN and DM, brain amyloidosis, and dementia. The coexistence of DM and HTN is associated with more profound cognition impairment than HTN alone.

This may be due to the overlapping of mechanisms that damage the central nervous system and deteriorate cognitive functioning. Further research is needed on this issue. The median M-ACE scale score was lower when HTN and DM coexisted than when HTN was diagnosed alone. Proper thickness of the intima–media complex at the level of the common carotid artery, which indicates normalization of arterial pressure, was bilaterally correlated with higher scores on the MMSE scale. Currently, educational level is considered to be a protective factor against the development of dementia in old age. This observation was confirmed in our study. Our study underscores the critical importance of early diagnosis and treatment HTN and DM. This can result not only in the prevention of strokes or myocardial infarctions but also in the maintaining of healthy cognition. Therefore, primary care units and other healthcare professionals should pay special attention to the diagnosis and effective treatment of HTN and DM.

## 6. Strengths and Limitations

The study was conducted in a rural area. Patients here have more contact with relatives and are more physically active, but the educational level is lower compared to the city’s inhabitants. People often eat more natural food produced in the local area.

In our study, we were not able to form a group of patients suffering from DM only, due to the small study population. Many patients initially qualified as DM only were diagnosed with HTN during the first appointment. It is necessary to confirm our observations among larger study groups. Further studies comparing rural and urban populations are needed. As it is a cross-sectional study in nature, causal relationships cannot be established.

This is a preliminary report.

## Figures and Tables

**Table 1:** The characteristics of the study group.

Parameter	Description	Total (%)	HTN&DM (%)	HTN (%)	HC (%)
N	Total	78	40 (47.1%)	18(21.2%)	20 (23.5%)
	women	50 (64.1%)	26 (65%)	11 (61.1%)	13 (65%)
Median age [years]		71	72.5	69	70.5
Professional activity	physical	53 (67.9%)	30 (75%)	11 (61.1%)	12 (60%)
	mental	25 (32.1%)	10 (25%)	7 (38.9%)	8 (40%)
Diet	none	21 (26.9%)	6 (15%)	3 (16.7%)	12 (60%)
	limited simple sugars	5 (6.41%)	3 (7.5%)	0	2 (10%)
	limited animal fats	11 (14.1%)	0	0	5 (25%)
	limited simple sugars and animal fats	41 (52.6%)	31 (77.5%)	9 (50%)	1 (5%)
Body mass	underweight	0	0	0	0
	normal range	9 (11.5%)	4 (10%)	0	5 (25%)
	overweight	31 (39.74%)	15 (37.5%)	9 (50%)	7 (35%)
	obese class I (BMI 30.0–34.9)	27 (34.6%)	13 (32.5%)	9 (50%)	5 (25%)
	obese class II (BMI 35.0–39.9)	9 (11.5%)	6 (15%)	0	3 (15%)
	obese class III (BMI ≥ 40)	2 (2.6%)	2 (5%)	0	0
Addictions	present tobacco smoking	13 (16.7%)	4 (10%)	4 (22.2%)	5 (25%)
	history of tobacco smoking	36 (46.15%)	19 (47.5%)	9 (50%)	8 (40%)
	regular alcohol consumption	20 (25.6%)	10 (25%)	5 (27.8%)	5 (25%)
Social conditions	good	78 (100%)	40 (100%)	18 (100%)	20 (100%)
Contact with close relatives	more than 3 times a week	72 (92.3%)	37 (92.5%)	16 (88.9%)	19 (95%)
	maximum 3 times a week	5 (6.4%)	2 (5%)	2 (11.1%)	1 (5%)
	loneliness	1 (1.3%)	1 (2.5%)	0	0
Accommodation	with close relative	69 (88.5%)	35 (87.5%)	15 (83.3%)	19 (95%)
	alone	9 (11.5%)	5 (12.5%)	3 (16.7%)	1 (5%)
	closed care facility	0	0	0	0
Education	basic	23 (29.5%)	13 (32.5%)	3 (16.7%)	7 (35%)
	professional	31 (39.7%)	18 (45%)	8 (44.4%)	5 (25%)
	medium	14 (17.9%)	4 (10%)	5 (27.8%)	5 (25%)
	post-secondary	5 (6.4%)	3 (7.5%)	1 (5.6%)	1 (5%)
	higher	5 (6.4%)	2 (5%)	1 (5.6%)	2 (10%)
High school certificate	yes	21 (26.9%)	8 (20%)	6 (33.3%)	7 (35%)

N—quantity, HTN—hypertension, DM—diabetes mellitus, HC—healthy controls.

**Table 2:** The parameters among NC and DC patients.

Parameter	Study Group	*n*	Median Value	Lower Quartile	Upper Quartile	95% CI Lower	95% CI Upper	Effect Size (Cohen)	*p* Value
Years of education	NC	54	11	10	13	0	3	0.717	0.015
	DC	23	10	8	11				
Duration of lipid disorders treatment [years]	NC	28	10	4	13	−9.500	2	−0.491	0.04
	DC	19	14	7	19				
IMC L [mm]	NC	54	0.9	0.8	1.1	−0.300	0	−0.691	0.005
	DC	22	1.1	1.0	1.2				
IMC R [mm]	NC	54	0.9	0.8	1.1	−0.300	0	−0.671	0.01
	DC	22	1.05	1.0	1.2				
Aβ1-42 [pg/mL]	NC	50	38.52	28.90	48.43	−4.561	−21.542	0.738	0.02
	DC	20	27.35	15.13	44.97				
Aβ42/40	NC	50	0.39	0.33	0.48	0.033	0.184	1.122	<0.000
	DC	20	0.29	0.25	0.36				
apoE [μg/mL]	NC	50	125.0	94.15	125.0	24.35	77.600	0.805	0.002
	DC	20	65.73	44.28	104.55				

NC—normal cognition group; DC—deteriorated cognition group; IMC L—intima–media complex thickness left side; IMC R—intima–media complex thickness right side; Aβ—beta-amyloid; apoE—apolipoprotein E.

## Data Availability

The original contributions presented in this study are included in the article/Supplementary Materials. Further inquiries can be directed to the corresponding author.

## Supplementary Materials

The following supporting information can be downloaded at https://www.mdpi.com/article/10.3390/neurolint17110180/s1. Figure S1: Years of education and M-ACE score range correlation; Figure S2: Duration of smoking and IMC R; Figure S3: Classification of intima-media complex thickness [mm] on the left side and MMSE score [points] among hypertensive patients; Figure S4: Intima-media complex thickness on the right side in study groups; Figure S5: Glycated hemoglobin and MMSE/M-ACE scores; Table S1: Effect sizes and confidence intervals considering disease profile; Table S2: Results of neurological, ultrasonographic and biochemical tests in different treatment groups; Table S3: Diabetes control and investigated factors among patients treated for diabetes (HTN&DM and DM).

## Abbreviations

The following abbreviations are used in this manuscript:

ABI L	ankle-brachial index left
ABI R	ankle-brachial index right
AD	Alzheimer’s disease
ADL	Activities of Daily Living
AGEs	advanced glycation end products
apoE	apolipoprotein E
APP	amyloid precursor protein
Aβ	beta-amyloid
Aβ42/40	plasma β-amyloid 1-42-to-plasma β-amyloid 1-40 ratio
BBB	Blood–brain barrier
BMI	Body Mass Index
CSF	cerebrospinal fluid
DC	deteriorated cognition group
DM	diabetes mellitus
DSST	Digit Symbol Substitution Test
EDTA	ethylenediaminetetraacetic acid
FDG-PET	18F-fluorodeoxyglucose positron emission tomography
HC	healthy controls
HDL	high-density lipoproteins
HTN	arterial hypertension
IADL	Instrumental Activities of Daily Living
IMC L	intima–media complex thickness left side
IMC R	intima–media complex thickness right side
LDL	low-density lipoproteins
M-ACE	Mini–Addenbrooke’s Cognitive Examination
MMSE	Mini–Mental State Examination
N	quantity
NC	normal cognition group
RAGE	advanced glycation end products receptor
RAS	renin–angiotensin system
ROS	reactive oxygen species
TC	total cholesterol
TG	triglycerides
UA	uric acid
