# Supplementary material for: The Cognitive Changes Among Patients over 65 Years of Age in a Rural Area—The Preliminary Report of Protective and Predisposing Factors
Source: Neurol Int. 2025 Nov 3;17(11):180. doi: 10.3390/neurolint17110180 (PMC12655145; doi:10.3390/neurolint17110180)
Supplement: Supplementary file 1 [file neurolint-17-00180-s001.zip › neurolint-3849342-supplementary.pdf]

Supplementary Materials (SM)

Table S1: Effect sizes and confidence intervals considering disease profile

Parameter	Study group	n	Median value	95% CI lower	95% CI upper	Effect size (Cohen)	p value
AM-42[pg/ml]	HTN&DM	36	32,8	-20,47	-0,82	-0,767	0,015
	HTN	18	43,3				
AM-42[pg/ml]	HTN&DM	36	32,8	-20,99	6,91	-0,545	0,047
	HC	17	41,4				
AM-42[pg/ml]	HTN	18	43,3	-8,12	16,64	0,135	0,314
	HC	17	41,4				
AM-42/AM-40	HTN&DM	36	0,32	-0,164	-0,02	-0,954	0,002
	HTN	18	0,40				
AM-42/AM-40	HTN&DM	36	0,32	-0,194	-0,043	-1,058	<0,000
	HC	17	0,47				
AM-42/AM-40	HTN	18	0,40	-0,13	0,07	-0,198	0,364
	HC	17	0,47				
M-ACE score [points]	HTN&DM	39	25	-6	-1	-0,922	0,006
	HTN	18	28				
M-ACE score [points]	HTN&DM	39	25	-2	-1,5	-1,303	<0,000
	HC	20	29				
M-ACE score [points]	HTN	18	28	-2	1	-0,486	0,287
	HC	20	29				
MMSE score [points]	HTN&DM	39	27	-3	0	-1,117	0,002
	HTN	18	29				
MMSE score [points]	HTN&DM	39	27	-3	-0,5	-1,102	<0,000
	HC	20	29				
MMSE score [points]	HTN	18	29	-1,5	1	-0,013	0,830
	HC	20	29				

Tables S2: Results of neurological, ultrasonographic and biochemical tests in different treatment groups

M-ACE - The Mini-Addenbrooke's Cognitive Examination, MMSE - Mini–Mental State Examination, IMC L – intima-media complex thickness left side, IMC R - intima-media complex thickness right side, ABI L - ankle-brachial index left, ABI R - ankle-brachial index right, N – quantity, HTN – hypertension, DM – diabetes mellitus, HC – healthy controls

Parameter	Description	Total	HTN&DM	HTN	HC
M-ACE range	*>25 points*	60	18	16	20
	22-25 points	13	12	1	0
	<22 points	10	9	1	0
MMSE range	*27-30 points*	62	25	18	19
	24-26 points	15	14	0	1
	<24 points	0	0	0	0
	no data	1	1	0	0
IMC L	*normal*	48	14	15	19
	above the norm	29	25	3	1
	no data	1	1	0	0
IMC R	*normal*	50	19	15	16
	above the norm	27	20	3	4
	no data	1	1	0	0
ABI L	*below the norm*	7	6	0	1
	normal	54	23	13	18
	above the norm	13	8	4	1
	no data	4	3	1	0
ABI R	*below the norm*	7	6	0	1
	normal	55	26	12	17
	above the norm	12	5	5	2
	no data	4	3	1	0
Treatment of lipid disorders	*no*	35	14	4	17
	yes	43	26	14	3
Treatment of uric acid disorders	*no*	67	30	17	20
	yes	9	8	1	0

Parameter	Study group	n	Median value	Lower quartile	Upper quartile	Quadrant deviation	p
Verbal fluency as a component of M-ACE [points]	**HTN&DM**	39	5,0	5,0	6,0	0,5	<0,000[a, b]
	**HTN**	18	7,0	6,0	7,0	0,5	<0,0000[a]
	**HC**	20	7,0	6,5	7,0	0,25	<0,0000[b]
Glycated hemoglobin [%]	**HTN&DM**	39	6,3	5,7	7,3	0,8	<0,000 [a, b]
	**HTN**	15	5,8	5,4	6,0	0,3	<0,000 [a]
	**HC**	18	5,5	5,4	5,8	0,2	<0,000 [b]
IMC L [mm]	**HTN&DM**	39	1,1	1,0	1,2	0,1	<0,000 [a, b]
	**HTN**	18	0,8	0,7	1,0	0,15	<0,000 [a]
	**HC**	20	0,8	0,7	0,9	0,1	<0,000 [b]
Uric acid [µmol/l]	**HTN&DM**	31	380,7	262,6	434,0	85,7	0,0243 [a]
	HTN	17	345,0	323,6	374,7	25,6	
	**HC**	18	280,9	256,0	315,0	29,5	0,0243 [a]
LDL [mmol/l]	**HTN&DM**	38	2,8	1,9	4,0	1,1	0,0028
	HTN	18	3,2	2,6	3,6	0,5	
	**HC**	19	3,96	3,4	4,6	0,6	0,0028
M-ACE [points]	**HTN&DM**	39	25,0	22,0	28,0	3	<0,000 [a, b]
	**HTN**	18	28,0	27,0	30,0	1,5	<0,000 [a]
	**HC**	20	29,0	28,0	29,5	0,75	<0,000 [b]
MMSE [points]	**HTN&DM**	39	27,0	26,0	28,0	1	0,0001 [a, b]
	**HTN**	18	29,0	28,0	30,0	1	0,0001 [a]
	**HC**	20	29,0	28,0	30,0	1	0,0001 [b]
Memory, as a component of M-ACE [points]	**HTN&DM**	39	5,0	3,0	6,0	1,5	0,0084 [a]
	HTN	18	6,0	5,0	7,0	1	
	**HC**	20	6,0	5,5	7,0	0,75	0,0084 [a]
Recalling as a component of MMSE [points]	**HTN&DM**	39	2,0	1,0	2,0	0,5	0,0117[a]
	HTN	18	2,5	2,0	3,0	0,5	
	**HC**	20	3,0	2,0	3,0	0,5	0,0117 [a]
TC [mmol/l]	**HTN&DM**	38	4,7	3,7	6,0	1,1	0,0196 [a]
	HTN	18	4,9	4,4	5,4	0,5	
	**HC**	19	5,8	5,0	6,5	0,7	0,0196 [a]

M-ACE - The Mini-Addenbrooke's Cognitive Examination, MMSE - Mini–Mental State Examination, IMC L – intima-media complex thickness left side, TC- total cholesterol, LDL - low-density lipoproteins, HTN – hypertension, DM – diabetes mellitus, HC – healthy controls. Statistics: Kruskal-Wallis' rank analysis for independent samples; [a; b] – corresponding p values

Figure S1: Years of education and M-ACE score range correlation

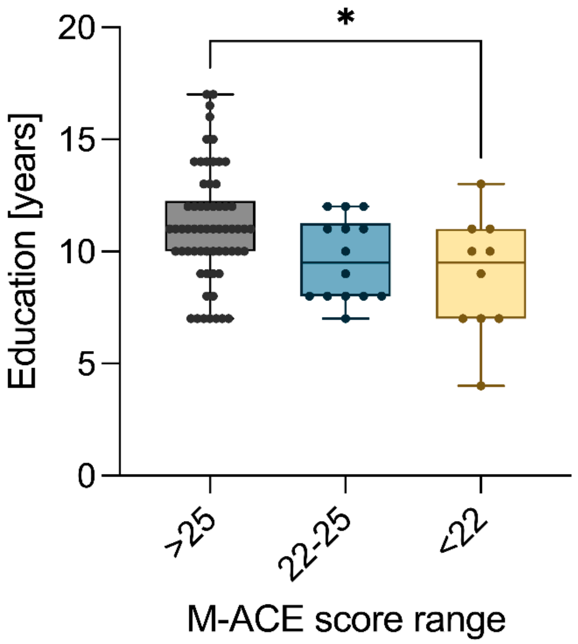

*Years of education and M-ACE score range, M-ACE - The Mini-Addenbrooke's Cognitive Examination. Statistics: Kruskal-Wallis' rank analysis for independent samples; * p < 0.05, ** p < 0,01, *** p < 0.001, **** p < 0.0001.*

Table S3: Diabetes control and investigated factors among patients treated for diabetes (HTN&DM and DM)

	Diabetes	N	Median value	Quadrant deviation	p
**TG [mmol/l]**	controlled	64	1,2	0,4	**0,004**
	uncontrolled	13	2,0	0,3	
**LDL [mmol/l]**	controlled	65	3,3	0,9	**0,037**
	uncontrolled	13	3,8	0,9	
**duration of diabetes treatment [years]**	controlled	31	8,0	3,5	**0,025**
	uncontrolled	12	11,0	5,8	

TG – triglycerides, LDL - low-density lipoproteins; Statistics: U Mann-Whitney test for independent samples

Figure S2: Duration of smoking and IMC R

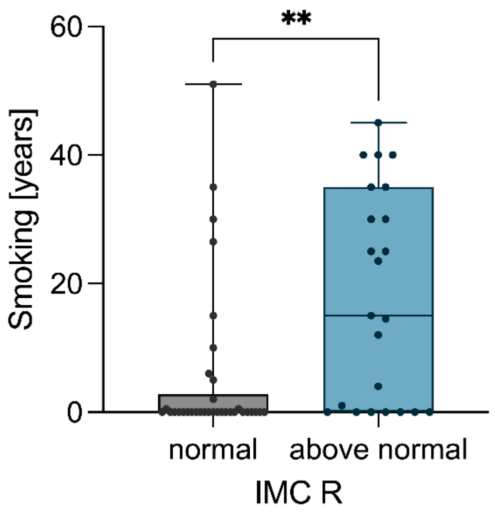

*Duration of smoking and classification of intima-media complex thickness on the right side among patients treated for arterial hypertension (HTN&DM and HTN), IMC R - intima-media complex thickness right side. Statistics: U Mann-Whitney test for independent samples, * $p < 0.05$, ** $p < 0{,}01$, *** $p < 0.001$, **** $p < 0.0001$.*

Figure S3: Classification of intima-media complex thickness [mm] on the left side and MMSE score [points] among hypertensive patients

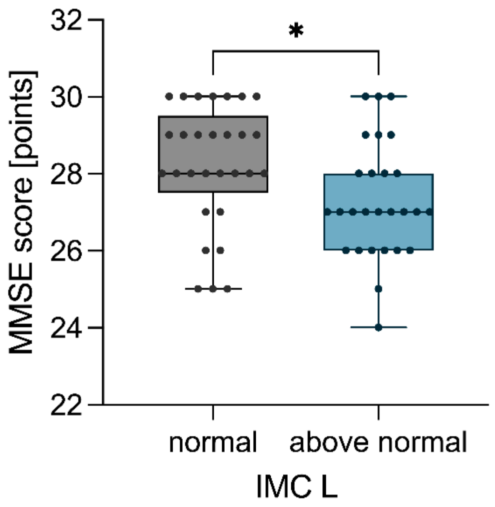

*Classification of intima-media complex thickness [mm] on the left side and MMSE score [points] among hypertensive patients (groups HTN&DM and HTN), MMSE - Mini–Mental State Examination, IMC L – intima-media complex thickness left side. Statistics: U Mann-Whitney test for independent samples, * p < 0.05, ** p < 0,01, *** p < 0.001, **** p < 0.0001.*

Figure S4: Intima-media complex thickness on the right side in study groups

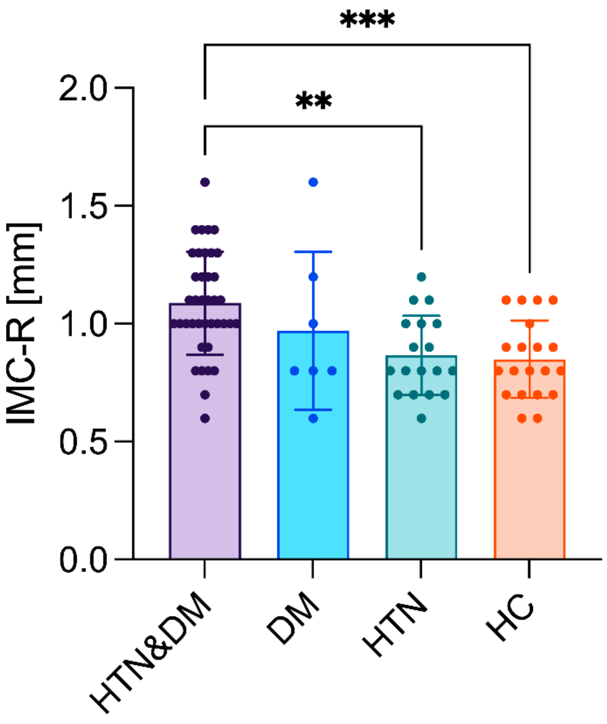

*Intima-media complex thickness on the right side in study groups, IMC R - intima-media complex thickness right side, HTN – hypertension, DM – diabetes mellitus, HC – healthy controls. Statistics: ANOVA for independent samples and post-hoc NIR test; * p < 0.05, ** p < 0,01, *** p < 0.001, **** p < 0.0001.*

Figures S5: Glycated hemoglobin and MMSE/M-ACE scores

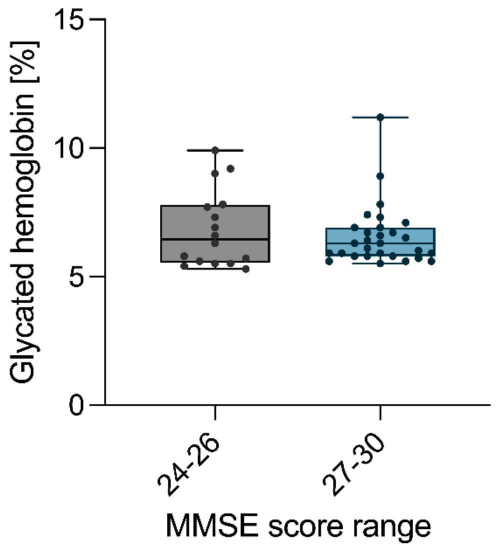

*Glycated hemoglobin percentage and MMSE score among patients treated for diabetes (groups 1 and 2), MMSE - Mini–Mental State Examination. Statistics: Not significant in U Mann-Whitney test * p < 0.05, ** p < 0,01, *** p < 0.001, **** p < 0.0001.*

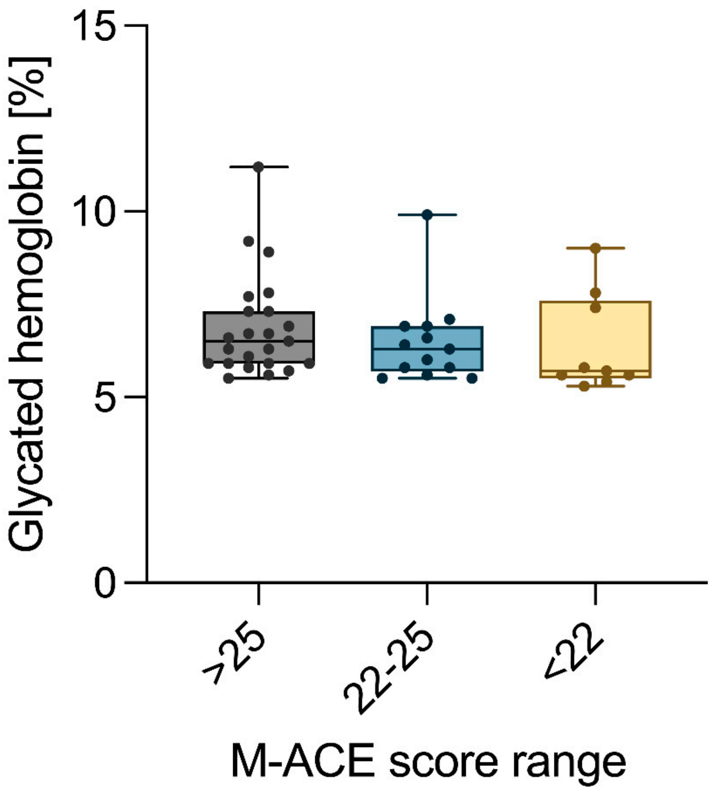

*M-ACE score and glycated hemoglobin percentage among patients treated for diabetes (groups 1 and 2), M-ACE - The Mini-Addenbrooke's Cognitive Examination. Statistics: Not significant Kruskal-Wallis' rank analysis for independent samples; * p < 0.05, ** p < 0,01, *** p < 0.001, **** p < 0.0001.*
